# Dermal Fillers: Do's and Dont's

**DOI:** 10.4103/0974-2077.63221

**Published:** 2010

**Authors:** Maya Vedamurthy, Amar Vedamurthy, KC Nischal

**Affiliations:** *Apollo Hospitals, Chennai, India*; 1 *Sri Ramachandra Medical College, Chennai, India*; 2 *Adichunchanagiri Institute of Medical Sciences, Karnataka, India*

**Keywords:** Dermal filler, training, documentation, statutory board approval

## Abstract

Dermal fillers are an important tool in the armamentarium of an aesthetic dermatologist in the management of ageing skin. A surge in the use of fillers has been witnessed due to increasing awareness among people, easy availability of fillers and increased enthusiasm amongst the dermatologists and plastic surgeons to use this modality. In this era of evidence-based medicine and litigations against doctors, Dermatologists should be vigilant about different acts of omission and commission in the use of fillers. This article briefly discusses the dos and don'ts with respect to dermal fillers.

## INTRODUCTION

Dermal fillers have revolutionized the field of cosmetic dermatology, as evidenced by the presence of a large number of products in the market. Even though fillers have been classified as a cosmetic device and have been FDA approved only for wrinkle management, they have been used for several other aesthetic and non-aesthetic indications too.[1]

It is crucial that dermatologists adopt safe practices in aesthetic dermatology and this article highlights the do's and dont's of filler practice to ensure successful outcomes for the patient and the physician.

## PRE-PROCEDURE CARE

### Know how the skin ages

Fillers are an armamentarium in the anti-ageing treatment. Though ageing is a generalized phenomenon, the process of ageing does not occur uniformly in all areas of the face. The subcutaneous fat of the face is not a single uniform layer but is compartmentalized.[2–4] Each individual compartment ages at a different pace in the same individual. Hence correction of the ageing face needs correction of these individual compartments separately.

### Know the fillers

There are a host of fillers now available in India. It is imperative to know about these fillers, their constituents, particles per mg, cross-linking, monophasic or biphasic nature, presence of any additive like lignocaine, and their shelf life before and after opening the filler.

### Know the technique

There are various methods of injecting the filler. Most popular is the linear threading method, either retrograde or antegrade. It is advisable that the beginners start with this technique than other methods like the depot or fern method which can be associated with lumps, if given inappropriately.

### Know how to know about fillers

Nowadays there are various opportunities to acquire knowledge regarding fillers. Theoretical knowledge can be acquired through textbooks, internet or journals.[5] Practical knowledge is best acquired by hands-on workshops or by working with seniors who are already practicing fillers. Workshops are conducted by national forums like Indian Association of Dermatologists, Venereologists and Leprologists (IADVL), Association of Cutaneous Surgeons of India (ACSI),[6] Indian Academy of Aesthetic Dermatology (IAAD)[7] and American Association of Aesthetic Medicine and Surgery (AAAMS);[8] training centres like KT Medical Aesthetic Training Centre,[9] Intraderm Ltd[10] and companies manufacturing fillers like Anteis[11] and which market fillers like Coherent Medical Systems.[12]

Initially, practice fillers under the direct supervision of a trained practitioner. It is better to practice fillers initially on people whom you personally know than on your patients. Do not venture into non-FDA approved indications. Anticipating any adverse effects, recognizing them promptly and institution of early rescue measures is as important as knowing fillers and administering them. When something is beyond your expertise, do not hesitate to take opinion of seniors.

### Know about the approval status of the filler

Fillers are considered as a medical device and hence there is a quality check conducted by various approval bodies in various parts of the world for their use. These approvals assert the authenticity and safety of products. Marketing of products is also easy if they have the certification by the appropriate bodies of that particular country. In Europe, Conformité Européenne (CE) marking is given for products complying with the laid standards.[13] In USA, an approval by Food and Drug Administration (FDA) is mandatory. The status of approval by FDA for any filler is widely accepted in most non-European countries and can be obtained online from the FDA's official website.[14]

Cost is a major issue in our country and this paves way for use of substandard materials to fulfill the patients' desire. New fillers from China,[15,16] Korea,[17] Syria,[18] Ukraine[19] and many other places are being introduced into the market, and in many instances full knowledge about the safety and approval status may be difficult to ascertain. Hence, dermatologists should restrain from using lesser known products. In such instances, the responsibility of treating physicians increases. It is important to note here the recommendation from the dermatosurgery taskforce guidelines of IADVL:[20]

Controlled data for the longevity of the filler materials published by the manufacturing company may not always be available. Both this and the fact that individual results may vary should be explained to the patient.

“Fillers from different countries are available in India and many of these may not have received approval from the drug authorities. It may not be always possible to use only FDA-approved fillers in our country. Controlled data for the longevity of the filler materials published by the manufacturing company may not always be available. Both this and the fact that individual results may vary should be explained to the patient. In view of these facts, full information about the filler and its approval status should be sought from the distributor, to satisfy oneself with full knowledge about the filler substance. Moreover, every country has its own approval systems and this should be taken into consideration.”

## ASSESSMENT OF THE PATIENT

### Assess the psyche of patients

This is very important as patients with unrealistic expectations will always end up dissatisfied which can demoralize the practitioners or jeopardize their practice. Know about their pain tolerance. Though fillers can be administered under topical anaesthesia, for patients with lower pain threshold, always perform the procedure under infiltrative anaesthesia.

### Medical assessment of patients

Check for the history of any medical illnesses and medications. Risk of bruising is more when patients have bleeding disorders, uncontrolled hypertension, or when on anticoagulants like aspirin, clopidogrel or warfarin. Always check for hypersensitivity to lignocaine. Anti-ds DNA antibodies cross-react with collagen and hence, collagen based fillers are contraindicated for patients with systemic lupus erythematosus.[21] Even though there are only occasional reports of any adverse events or suboptimal results, caution has been advised while injecting hyaluronic acid-based fillers derived from Streptococcus species in patients with any previous streptococcal disease.[22,23] Finally, check for any signs of inflammation in the area to be treated. Active inflammation leads to degradation of the filler.

## AESTHETIC ASSESSMENT OF PATIENTS

Aesthetic evaluation of the face must first consider the goals of the patient: does the patient want to restore the old original look or does the patient want a new and different look? The assessment of the area to be treated should be done with the patient in an upright position. Gravitational force alters the defects or grooves. It is also important to identify any asymmetry and make the patient aware of it before starting the procedure. Proper judgement can be done by taking a photograph and studying it. As described above, check for the exact Part of face to be treated.

## CHOOSING THE RIGHT FILLER

At present, there is a wide range of filler materials available in the country that vary in source, longevity, site of deposition and cost. One has to choose the right tool for the right job. This is based on the indication and site of placement. The medical condition of the patient should always be considered while using the fillers.

Choice of fillers depends upon various factors:

- Defect to be corrected: For volumizing or augmentation, denser fillers are required; for superficial defects, lighter molecules are preferred.
- Longevity desired: For prolonged effects, permanent fillers are preferred.
- Material: Collagen-based fillers have to be pre-tested before administration.[2,24]

However, combining different filler materials at the same site in the same session is not recommended due to inherent complications that could arise as a result of the materials.[4,20]

## DOCUMENTATION

### Obtain informed consent

It is very important that the patient is educated about the filler, technique of administration, outcome, side effects, post-procedure care, need for maintenance and any other additional procedures required to achieve optimum results. A handout on these aspects can be given to patients for reference. Consent has to be signed after counselling and adequate briefing of the patient.

### Document the findings

Well-focused, pre-treatment photographs should be taken not only for a better assessment of defects, but also for legal purposes. However, consent has to be taken specifically for facial photographs where identity could be revealed. Images are better taken without zooming in a well-lit room and without flash. Document any asymmetry present between two sides.

### Discuss the cost of the treatment

The type of filler, quantity required and the cost involved should be discussed before starting the procedure. Touch-up session and any additional requirement of the filler during the touch-up and the probable expense that could be incurred have to be briefed.

Longevity of results is a crucial issue in the use of fillers. Patients should always know fully the duration of results expected for proper planning. As stated above, controlled data for the longevity of the filler materials may not always be available. Both this and the fact that individual results may vary should be explained to the patient.

## INTRA-PROCEDURE CARE

### Preparing the area for filler administration

Strict asepsis has to be maintained during filler administration. The area to be treated has to be disinfected with the spirit-povidone iodine-spirit method. Sterile cotton and gauze has to be used.

### Anaesthesia

Any medical intervention has to be as painless as possible, more so when an enhancement procedure like filler procedure is being done. However, procedures to alleviate pain should not interfere with treatment outcomes. This is frequently encountered when an infraorbital nerve block is used for filling the nasolabial groove. Lignocaine injected in the infraorbital region distorts the local anatomy and may result in suboptimal correction. The quantity of lignocaine required and the resultant distortion can be reduced by using 1 ml of lignocaine 2% administered using an insulin syringe. The application of topical anaesthetic creams such as EMLA can improve the hydration of the skin and make fine lines imperceptible leading to imperfect correction. In such situations, choosing a filler premixed with lidocaine or a regional nerve block would be a suitable option. Inadequate anaesthesia achieved by EMLA can be compensated by the use of ice-packs. When employing linear threading or even depot technique, the number of pricks and hence the pain can be minimized by using long needles, of 1.5 inches.[6,25] Always check for lignocaine hypersensitivity for every patient.

### Technique

The right technique of administration has to be used for the filler being injected and for the site where it is being deposited.

#### Adhere to the right plane

If a filler that should be placed superficially is injected deep, it just diffuses in the deeper plane requiring larger volumes to fill the defect and also results in faster resolution. Denser fillers when injected superficially lead to lumpiness and blanching. If lumping is seen, moulding should be done immediately on recognizing it till the lump is flattened and the blanching is reversed. Though moulding is best done immediately, it may be done till 2 weeks after injecting the filler.

#### Method of administration

As an alternative to percutaneous injections for the supraperiosteal or subcutaneous deposition of fillers for cheek augmentation, an intra-oral approach can be employed. However, there is no consensus on the choice of technique in this regard.[26]

#### Correcting the defect

Check for asymmetry and individualize the amount required based on this. Undercorrection is always better and safer than overcorrection. Overcorrection can be rectified by injecting hyaluronidase for hyaluronic acid-based fillers[27,28] or aspirating by a wide-bore needle or incising and extracting the excess amount or with lasers.[29]

#### Retain the batch or lot number sticker

The batch number sticker provided with the filler should be pasted in the patient's consent form. Untoward effects arising from manufacturing discrepancies can be traced back by the batch number.

#### Documentation of the procedure done

The name of the filler and quantity injected in different areas should be mentioned in a chart which contains a sketch of the body part treated. This is not only of relevance for follow-up but also very important if the patient is being considered for any other anti-ageing procedures like laser, radiofrequency, etc. (refer to the text below).

## POST-PROCEDURE CARE

Post-procedure care plays a vital role in the achievement of optimal results and hence it is essential to educate patients on this aspect.[8,30]

### Post-procedure instructions

The patient is advised to refrain from massaging the injected area for 2 weeks and exposing to extremes of temperatures like sauna or skiing. Counsel the patient that if any bruising occurs; it is likely to be transient. Follow-up visits for touch-up treatment should be after 2 weeks.

## STORAGE OF THE REMAINING FILLER

Storing of the leftover filler material for subsequent use for the same patient is a much debated topic. Bellew *et al*., studied the sterility of non-animal stabilized hyaluronic acid gel syringes stored for 2–9 months at room temperature after patient injection. They could not culture any bacterial growth (aerobic as well as anaerobic) from any of the samples.[31] Similar studies done with injectable collagen also opined favourably on the reuse of the stored collagen.[32,33]

Hence, syringes containing filler can be preserved for 4 weeks in a refrigerator (do not freeze the filler!). The needle is detached and the rubber cap fitted back. The name of the patient and the date of the procedure done are mentioned on the packing.

### Complications

Dermatologists should be aware of all the potential complications that could arise either by the injection itself or the implanted material.[7,34] Special precautions need to be carried out especially on the glabella, periorbital area and tip of the nose.[1,35] Strict asepsis throughout the procedure is advised to avoid infections. One should be able to manage complications without any adverse sequelae, or be able to refer the patient to an appropriate centre for further management. Proper patient selection, proper technique and right choice of fillers would minimize the incidence of complications.

## COMBINING THE FILLER WITH OTHER PROCEDURES

In many patients, different anti-ageing treatments may need to be combined and hence proper knowledge of the effects of such combinations is essential.

### Fillers and lasers

As the chromophore for lasers used for skin rejuvenation is water, there is a theoretical risk of dissolution of hyaluronic acid-based fillers, when such lasers are administered to treated areas. Goldman *et al*., administered 1320-nm Nd:YAG laser, 1450-nm diode laser, monopolar radiofrequency and/or intense pulsed light immediately after injecting hyaluronic acid-based dermal fillers (Restylane™) into the nasolabial groove. They found no reduction in the clinical outcome.[36] However, larger studies are essential to authenticate this observation.

In patients with prominent photoageing, De Maio suggests that laser resurfacing should be done first and then the filler procedure, once the process of collagen remodelling has been completed. He has also opined that nasolabial grooves become shallower due to overall tightening of skin. However, aggressive resurfacing is fraught with risk of dyspigmentation in darker skin types.[5]

### Fillers and chemical peels

All chemical peels elicit some amount of inflammation and this inflammation has a theoretical risk of degradation of the filler. De Maio and Rzany opine that as the inflammation elicited by superficial chemical peels is not significant, superficial peels can be done immediately after filler administration. They advise to defer medium-depth peels, namely trichloroacetic acid till the post-peel erythema fades or till collagen remodelling is completed (probably in 1–2 weeks).[5] This opinion is in contrast to that of Deprez who has found no such association.[37] In the absence of proper studies, it is difficult to arrive at a consensus on this aspect.

### Fillers and botulinum toxin

Combining fillers with botulinum toxins is a new rejuvenation paradigm.[38] Since the hyperactive or hypertonic muscles play a prominent role in producing wrinkles, it is better to relax the muscles first with the botulinum toxin, and later administer fillers after 2 weeks. However, for the nasolabial groove, fillers are injected first and then the botulinum toxin is injected.[5]

### Fillers and radiofrequency

Radiofrequency is one of the common modalities employed for non-ablative skin rejuvenation. The efficacy of a filler (both hyaluronic acid- and non-hyaluronic acid-based fillers) was found unaltered when non-ablative radiofrequency was performed over areas treated with the filler.[36,39] However, more data are needed to draw concrete conclusions on this aspect.[40]

### Fillers and aesthetic plastic surgery

Plastic surgery for facial contouring and other aesthetic indications can be supplemented by fillers. Fillers have the advantage of achieving finer corrections.

## SUMMARY

There has been a rapid expansion in the use of dermal fillers in dermatology–aesthetic practice. While using fillers, all young dermatologists would do well to remember the advice of William Osler: ‘Be not the first to try the new nor the last to lay aside the old’.
